# Global dynamics of newly constructed oligonucleosomes of conventional and variant H2A.Z histone

**DOI:** 10.1186/1472-6807-7-76

**Published:** 2007-11-08

**Authors:** Amutha Ramaswamy, Ilya Ioshikhes

**Affiliations:** 1 Department of Biomedical Informatics, The Ohio State University, Columbus, Ohio, USA; 2 Department of Biomedical Informatics, Department of Molecular and Cellular Biochemistry and Davis Heart and Lung Research Institute, The Ohio State University, Columbus, 3172c Graves Hall, 333 West 10th Avenue, Columbus, Ohio 43210, USA

## Abstract

**Background:**

Complexes of nucleosomes, which often occur in the gene promoter areas, are one of the fundamental levels of chromatin organization and thus are important for transcription regulation. Investigating the dynamic structure of a single nucleosome as well as nucleosome complexes is important for understanding transcription within chromatin. In a previous work, we highlighted the influence of histone variants on the functional dynamics of a single nucleosome using normal mode analysis developed by Bahar et al. The present work further analyzes the dynamics of nucleosome complexes (nucleosome oligomers or oligonucleosomes) such as dimer, trimer and tetramer (beads on a string model) with conventional core histones as well as with the H2A.Z histone variant using normal mode analysis.

**Results:**

The global dynamics of oligonucleosomes reveal larger amplitude of motion within the nucleosomes that contain the H2A.Z variant with in-planar and out-of-planar fluctuations as the common mode of relaxation. The docking region of H2A.Z and the L1:L1 interactions between H2A.Z monomers of nucleosome (that are responsible for the highly stable nucleosome containing variant H2A.Z-histone) are highly dynamic throughout the first two dynamic modes.

**Conclusion:**

Dissection of the dynamics of oligonucleosomes discloses in-plane as well as out-of-plane fluctuations as the common mode of relaxation throughout the global motions. The dynamics of individual nucleosomes and the combination of the relaxation mechanisms expressed by the individual nucleosome are quite interesting and highly dependent on the number of nucleosome fragments present in the complexes. Distortions generated by the non-planar dynamics influence the DNA conformation, and hence the histone-DNA interactions significantly alter the dynamics of the DNA. The variant H2A.Z histone is a major source of weaker intra- and inter-molecular correlations resulting in more disordered motions.

## Background

In a previous work, we described the influence of histone variants on the functional dynamics of a single nucleosome using normal mode analysis [1]. Knowledge of the dynamics of nucleosome complexes with conventional histones or histone variants would enrich our understanding of the role of chromatin in regulating or modifying gene transcription [2-5]. Significant advances have been made to elucidate the structure of compact nucleosome complexes in specific promoter regions where chromatin structure should be remodeled during transcription.

**Figure 1:**
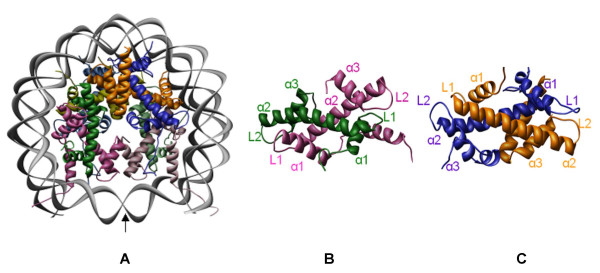
**The architecture of the nucleosome**. **(A) **The conformation of variant nucleosome constructed using the PDB file 1F66 deposited by Suto et al. [14]. Histones H3 (pink), H4 (green), H2A.Z (blue) and H2B (orange) are wound by the superhelical DNA (gray) in the nucleosome and the heterodimers H3-H4 **(B) **and H2A-H2B **(C) **are shown separately for clarity. The symmetry of the nucleosome is indicated by an arrow (named as dyadic axis).

The active role of chromatin in the regulation of gene activity implies conformational flexibility of the basic chromatin structural unit, the nucleosome. The nucleosome is composed of 146 bp of DNA wrapped around an octameric histone core consisting of two copies of core histones H2A, H2B, H3 and H4 [6-8]. In the histone core, the H3 and H4 associate into two heterodimers and further assemble into a tetramer of (H3-H4)_2 _whereas the histones H2A and H2B form independent heterodimers 2(H2A-H2B) [9-12]. Each core histone possesses two functional domains: a "histone fold" motif (involved in both histone-histone and histone-DNA interactions within the nucleosome) and the NH_2_- as well as COOH- terminal "tail" domains (which contain sites for posttranslational modifications such as acetylation, methylation and phosphorylation) [13]. The histone fold possesses three alpha helices (α1, α2, α3) connected by two loops (L1, L2). The nucleosome DNA is wrapped around the histone octamer and establishes stable interactions with histones, where the two L1L1 sites and one α1α1 site of the histone fold motif directly contribute to the DNA-histone interactions. The nucleosome possesses a dyad symmetry with the symmetry axis passing through the octamer and intersecting the DNA at the midpoint of the bound DNA sequence. Fig. 1 shows the structure of variant nucleosome constructed using the PDB file 1F66 (Fig. 1A) deposited by Suto et al. [14] and the heterodimers formed by H3-H4 (Fig. 1B) and H2A.Z and H2B histones (Fig. 1C). Nucleosomes are arranged like "beads on a string" model, in which they are separated by a short sequence of DNA called linker. Such formation of nucleosomal complex is stabilized by the binding of a fifth histone (named a linker histone, H1) with its globular domain bound to the octamer near the point where the DNA enters and exits the nucleosome (dyad region) and seals the two full turns (approximately 166 bp) of DNA around the octamer [15]. The globular domain of the linker histone is a three-helix bundle that has structural homology to helix-turn-helix DNA-binding proteins, which simultaneously binds to the two DNA duplexes of the nucleosome [16,17]. Nucleosomes, connected by about 20 to 60 bp of linker DNA, form a 10-nm "beads on a string" array that can be compacted further into a 30-nm chromatin fiber [18-20]. The nucleosome particles can be modified in composition and structure by the incorporation of variant histones, which change the local chromatin structure to facilitate cellular processes such as transcription or development [21,22].

Variants have been described for several classes of histones, and one of the best-studied examples is the Z variant of H2A (i.e. H2A.Z). H2A.Z is a H2A subtype that has been identified in organisms as diverse as *Saccharomyces cerevisiae*, *Tetrahymena*, *Drosophila*, and *Homo sapiens *[23-26]. This protein displays 60% homology with H2A and 90% homology between species. Mutagenic assays have demonstrated that H2A.Z is essential for development in yeast [27] and for survival in *Tetrahymena *[28] and *Drosophila *[29,30]. The nucleosomes containing H2A.Z impact transcription activation within chromatin. Several pioneering studies, a few of which are mentioned here, have reported the importance and the functional diversity of the nucleosomes with H2A.Z. Suto et al. have characterized the histone-DNA and histone-histone interactions within the nucleosome core particle containing the variant H2A.Z histone [14]. Jackson and Gorovsky demonstrated that H2A.Z genes are functionally conserved and distinct from the genes of the major H2A sequence variants [27]. H2A.Z has been observed to be located at yeast promoters and to display a redundant role with ATP-dependent nucleosome remodeling complexes [31]. A review on the functional diversity of histone variants was reported by Brown [32]. Evolutionarily conserved variant histone H2A.Z has recently shown to regulate gene transcription in *Saccharomyces cerevisiae *[33]. H2A.Z modulates functional interactions with transcription regulatory components, thus helping to poise chromatin for gene activation [34]. H2A.Z alters the nucleosome surface to promote HP1α-mediated chromatin fiber folding [35]. The H2A.Z/H2B dimer is unstable compared to the dimer containing the major H2A isoform [36]. Studies on H2A.Z are expanding due to the importance of the H2A.Z variant histone in both positive and negative gene transcription.

Recent publications show controversial observations of the role of H2A.Z variants. Ausio group showed that histone variant H2A.Z has a destabilizing effect on both intranucleosomal histone-histone interactions and the internuclosomal structure [37]. In contrast, Fan et al. showed that H2A.Z facilitates the intramolecular folding of nucleosome arrays while simultaneously inhibiting the formation of highly condensed structures that are a result of intermolecular association [38]. In addition to these puzzles, a new Fluorescence Resonance Energy Transfer (FRET) approach revealed that the H2AZ variant histone stabilizes the histone octamer within the nucleosome [39]. Continuing in the same line, Placek et al. declared that the H2A.Z/H2B dimer is the least stable histone fold characterized to date, while H2A/H2B appears to be the most stable [36]. On the contrary, a more recent study by the Ausio group reinforced the role of H2A.Z in the stabilization of nucleosome [40]. Recently, Greaves et al. showed that H2AZ is a core component of heterochromatin needed for its integrity and proper sister chromatid cohesion [41]. A few more articles also explain the contrasting functions of H2A.Z and the nucleosome stability [42-45]. In order to identify the structural dynamics of nucleosome with variant histone H2A.Z within the above context, we dissected the global dynamics of the nucleosome complexes with conventional as well as variant histone H2A.Z to rationalize the stabilizing/destabilizing role of H2A.Z histone over the global dynamics of the nucleosome in normal mode analysis.

Traditional molecular simulation by full-atomic force fields [46,47] is a very time-consuming method for exploring dynamics of the nucleosome (~50,000 atoms). Recently, Sharma et al. described a multiscale modeling of nucleosome dynamics using discrete molecular dynamics [48]. The normal mode analysis (NMA) [49,50] is an efficient yet physically meaningful approach. It assumes that the molecular potential energy has quadratic shape and that the generalized eigenvalue problem is solved to obtain the analytic description of the motion. The eigenvalues give the frequencies of the vibration whereas the eigenvectors represent the corresponding motion. In addition, positional fluctuations or time correlation functions can be calculated. Because of the difficulty of handling larger biomolecular systems, the simplified NMAs based on the elastic network model [51-56] were developed and successfully applied to some molecular systems [55-58]. The Gaussian Network Model (GNM) [52,53] and Anisotropic Network Model (ANM) [56], proposed by Bahar and collaborators, have been extensively used to predict the structure and direction of residues as explained in the Methodology section [59-62].

This article addresses the global dynamics of the nucleosome complexes (oligonucleosomes) with conventional histones as well as variant H2A.Z and is an extension of our previous work on the structural dynamics of the single nucleosome [1].

## Results and Discussion

The global dynamics of the nucleosomal complexes have been studied systematically starting with the analysis of the dimer and extending up to the tetramer. The specified larger cutoff distances account for the complete protein-DNA interactions. Interpretations of global modes are made by comparing the theoretically-generated thermal fluctuations with the experimentally-observed thermal fluctuations of the residues.

### Validation of the constructed model of 1EQZ, 1F66 dimers with 1ZBB dimers

**Figure 2:**
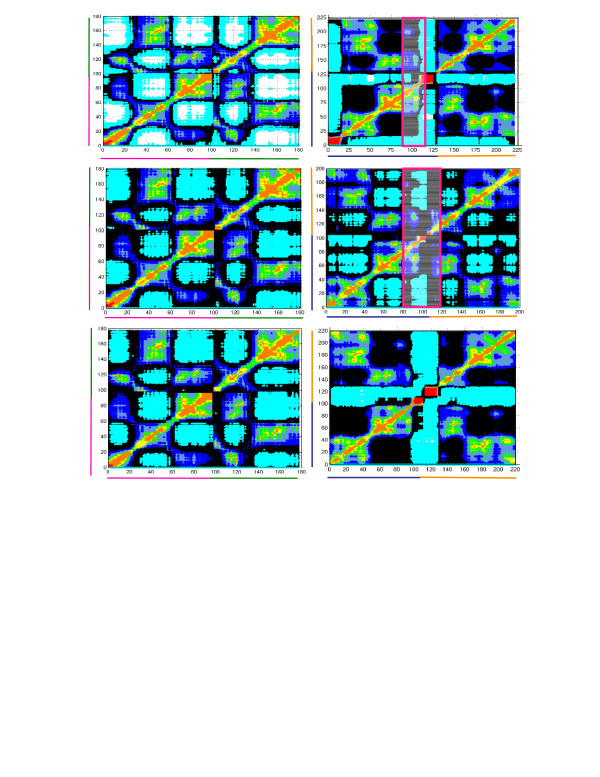
**Correlation between the motions of residues of the first nucleosome in the dimer**. Cross correlations between the motions of residues of dimers H3-H4 (A) and H2A-H2B (B) in the first nucleosomes in dimer 1EQZ (I), 1F66 (II) and 1ZBB (III) crystal structures. The uncorrelated regions (colored black) separate the correlated (where the amplitude increases from blue to red) and anti-correlated regions (colored cyan).

**Figure 3:**
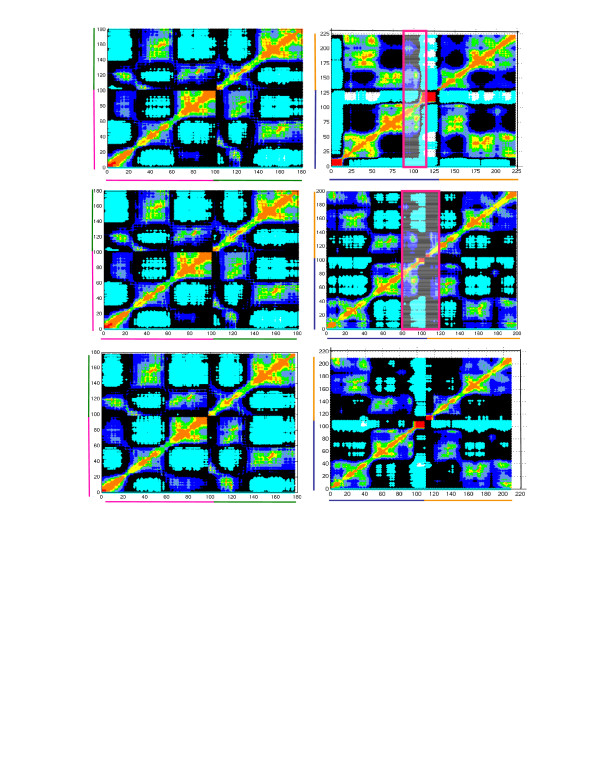
**Correlation between the motions of residues of the second nucleosome in the dimer**. Cross correlations between the motions of residues of dimers H3-H4 (A) and H2A-H2B (B) in the second nucleosomes in dimer 1EQZ (I), 1F66 (II) and 1ZBB (III) crystal structures; the amplitude of interactions follow the same color coding as Figure 2.

The comparison between the inter- and intra- domain motions of our model dimer and the dimer established by Schalch et al. (selected from the experimentally resolved tetromeric conformation) [63], confirms that the modeled nucleosome dimer of conventional and variant histones replicates the experimentally observed dimer conformation. Fig. 2 and Fig. 3 describe the correlations between the motions of residues of dimers H3-H4 (A) and H2A-H2B (B) of 1EQZ (I), 1F66 (II) and 1ZBB (III) crystal structures in the first (Fig. 2) as well as second (Fig. 3) nucleosome of the dimer. The cross-correlations between the residues are evaluated from the off-diagonal elements of Ã^-1^[52]. The uncorrelated residues (colored black) distinguish the correlated domain regions (colored from blue to red where the stronger correlation corresponds to the red region) and anti-correlated domain regions (colored cyan) in the nucleosome. In the case of H3-H4 dimers, the crystal structures 1EQZ, 1F66 and 1ZBB show a similar pattern of correlated and anti-correlated domain motions in both nucleosomes of the dimers. The correlated and anti-correlated domain motions in H2A-H2B and its variant dimer H2A.Z-H2B show a distinguished correlation pattern due to the presence of variant histone that influences the nucleosome dynamics. The intended goal of our comparison is to validate our model with experimental work. Therefore, details regarding the influence of variant H2A.1 and H2B.1 (in 1ZBB) over the global dynamics are beyond the scope of this article.

The correlation map of dimers H3-H4 and H2A.1-H2B.1 would be different for the two nucleosomes present in 1ZBB if the second nucleosome had influence over the dynamics of the first nucleosome and vice versa. However, it is clear from Fig. 2 and Fig. 3 that the dynamics of the first and second nucleosomes possess similar domain interactions in 1ZBB. The comparative analysis of cross-correlations shows that the constructed dimers 1F66 and 1EQZ have a similar correlation pattern as the nucleosome dimer 1ZBB[63]. This observation reinforces that the model dimer and the experimentally proven conformation express similar inter- as well as intra- domain motions. Therefore, the modeled conformation is similar to the experimentally observed conformation, thus supporting our method of modeling the nucleosome complexes. This approach enabled us to model the complexes such as dimer, trimer and tetramer. Further extension of this approach would provide additional insights about chromatin structure/biology.

### Inter- and intra-domain motions of the handshake motif in nucleosome dimer

Fig. 2 and 3 map the correlations between the residue motions of dimers H3-H4 and H2A (H2A.Z in 1F66 and H2A.1 in 1ZBB)-H2B (H2B.1 in 1ZBB) for the three examined structures, labeled I-III. In H3, the α2 helix acts as a global hinge-bending center for the anti-correlated motions expressed between the individual DNA binding loops (L1, L2), where short helices are correlated with the neighboring loops (L1 or L2 respectively). Similarly, H4 possesses two highly anti-correlated domains from N- to C- terminal, where the central residues of the helix α2 play a role of hinge domain. In this handshake motif, the two regions: (i) the loop L2 and α3 of H3 and the loop L1 and α1 of H4, (ii) L1 and α1 of H3 and L2 and α3 of H4, possess a highly correlative interaction within themselves and move in opposite directions. The dynamic N-terminus of H4 plays a vital role in gene silencing [64]. In the presence of variant histone H2A.Z, the interactions in the cooperative and anti-cooperative regions of H3-H4 motif are weakened.

Nearly the entire H2A-H2B motif possesses correlated motions except for the C-terminal of H2A (as it inserts into H3-H4 motif) and the N-terminal of H2B. The presence of H2A.Z variant disrupts the coherent motions between the residues resulting in the anti-correlation of the uncorrelated residues. The presence of H2A.Z introduces significant differences in intra- and inter-molecular correlations. In particular, the presence of H2A.Z decreases the coupling between (i) its docking domain and the helix-loop α_1_L1, and (ii) the loop-helix L2α_3 _of H2B (regions highlighted in the magenta boxes). Such disruptive behavior of H2A.Z supports the experimentally observed chromatin-destabilizing role of H2A.Z [37]. The histone H2B also exhibits H2A dependent inter- and intra-correlated domain motions as the cooperation of the motions decreases with the increasing H2A.Z mobility.

In general, the correlations between the motions of the handshake H2A-H2B (or H2A.Z-H2B) motives of the nucleosome dimer are similar to the correlation of H2A-H2B (or H2A.Z-H2B) motives of a single nucleosome and are also the same for H3-H4. With this argument, we have not extended the same analysis on the variant trimer as well as the tetramer. Because our main focus is on the analysis of the influence of H2A.Z over the dynamics, the interactions generated in H2A.1-H2B.1 motif of 1ZBB conformation have not been considered.

### Global dynamics of nucleosome complexes

**Figure 4:**
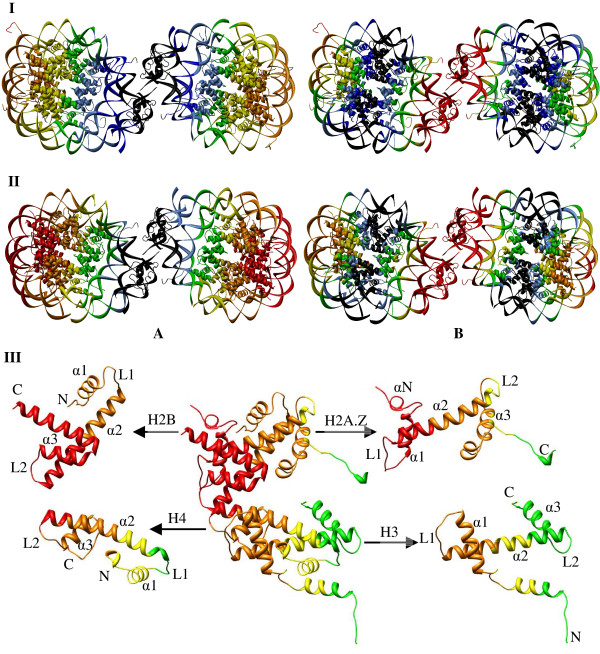
**Color-coded representation of the dynamics of the nucleosome dimer**. The color-coded ribbon diagram shows the dynamics of the dimers 1EQZ**(I) **and 1F66**(II) **in the first **(A) **and second **(B) **global modes. The amplitude of fluctuation is graded from black to red (i.e., from the rigid to the flexible domains, respectively). For an easy understanding, the closest view of the color coded monomeric histone tetramer of the left side nucleosome in 1F66 dimer in the first global mode is shown (middle) in the subdiagram **III **and the secondary structures of the four histones (H3, H4, H2A.Z and H2B) are labeled individually.

The dynamics of the nucleosome dimer with conventional and variant H2A.Z histone is shown in Fig. 4. Panel I represents the global dynamics of the nucleosome dimer with conventional histones and panel II is for the dimers of nucleosome with variant histone H2A.Z in the first (A) and second (B) slowest modes. The amplitude of fluctuation is graded from black (the lowest) to red (the highest) in order to show the variation in the amplitude of dynamics across the region from the rigid to the dynamic domain.

Analysis of the rigid domains of the nucleosome dimer with conventional histones (Fig. 4IA) reveals that almost half of the nucleosome encompassing the regions from the α3 helix and the L2 of H3 that interact with the DNA, linker DNA, and linker histone (regions colored from black to blue) are constrained by dynamics. The N terminal domain of H3, one of the major as well as important sites for transcription modification, is highly dynamic. In the case of the nucleosome with variant H2A.Z (Fig. 4IIA), the rigid domains are defined only by the linker DNA and linker histones. It is observed that the conventional histones restricted to move in the global modes are released for free motion in the presence of the variant H2A.Z histone. Although the dimer formed by H2A-H2B shows pronounced dynamics (colored from yellow to orange), the amplitude of dynamics for the dimer H2A.Z-H2B is even stronger (colored from orange to red) revealing the enhancement in the amplitude of dynamics from the presence of variant H2A.Z histone [43]. This supports the experimental observation of unstable H2A.Z/H2B heterodimer compared to the dimer containing the major H2A isoform [36]. Markedly stronger dynamics due to the presence of H2A.Z may drive the histone proteins to actively participate in the dimer global mode. Therefore, the constrained or frozen regions in the conventional nucleosome are dynamic in the nucleosome with H2A.Z variant.

Consequently, it is clear that the nucleosome dimer within the global mode, undergoes a highly dynamic motion within the regions encompassed by the linker DNA and the linker histone H1 as the hinge domains for the free dynamics of the dimer (Fig. 4). In our previous analysis, the monomeric nucleosome expressed highly symmetric dynamics with respect to the dyad axis in the global mode. In the second slowest mode, the dynamics have been identified as conjugate to that of the first slowest mode of motion, i.e., the central region of the nucleosome perpendicular to the dyad axis is highly constrained (rigid). When we compare this observation with the current observation of the nucleosomes in the dimer, it is very interesting to note that the global dynamics of the two single nucleosomes does not reflect the dynamics pattern observed for the monomeric nucleosome in its global mode. Here, in the global mode, the nucleosomes are highly dynamic while keeping the linker DNA/histone as the rigid domains.

The dynamics of the nucleosome dimer in the second slowest mode is comparatively constrained along the axis conjugate to the nucleosome dyad (Fig. 4B). Unlike the first global mode, the linker DNA and the linker histone are highly dynamic (colored red), and the regions of the H2A-H2B dimer interacting with the peripheral DNA sequences (Fig. 4IIB) are also in motion. In the case of nucleosome with H2A.Z variant, the DNA-(H2A.Z-H2B) interacting regions (α2-L2-α3-αC regions of H2B and α2-L1-α1 of H2A.Z) express stronger dynamics along with the interacting DNA segment than that of histone H2A. Furthermore, the dynamics of linker DNA/histone are more pronounced in the nucleosome dimer with H2A.Z. The vital observation is, in the second slowest mode, the nucleosome dimer reinforces the conjugate dynamics as that of the second global mode of the single nucleosome established by Ramaswamy et. al. [1]. When compared to the relaxation of the nucleosome dimer in the slowest global mode, the nucleosome dimer with conventional histones is almost stationary in the second slowest mode.

The dynamics of a nucleosome with conventional and variant histone are comparable, but the amplitude of dynamics is significantly higher for the dimer of variant nucleosome than that of the conventional nucleosome dimer (Fig. 4). The rigid domains observed in the second slowest mode are wide spread compared to the second slowest mode of nucleosome with variant H2A.Z histone (Fig. 4B). The variation in the amplitude of nucleosomal dynamics with the presence of H2A.Z variant histone is elaborated in our previous study [1]. The distinct behavior of linker histone in both slow modes may reinforce the observation of its ability to control the mobility of nucleosomes on DNA [65].

**Figure 5:**
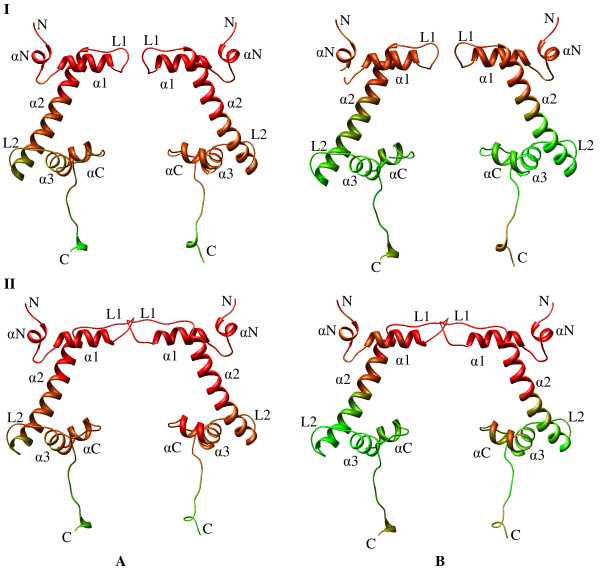
**Interaction of H2A and H2A.Z dimers in the nucleosome**. The color-coded ribbon diagram of the dynamics of H2A **(I) **and H2A.Z **(II) **dimers in the first **(A) **and second **(B) **global modes.

The observed influence of the variant histone over the dynamics of the nucleosome prompted us to look into the dynamics of variant H2A.Z histone in comparison to the conventional H2A histone. Fig. 5 shows the color-coded ribbon diagram of the dynamics of H2A (**I**) and H2A.Z (**II**) dimers in the first **(A) **as well as second **(B) **global modes. The secondary structures are labeled for easy understanding. We know that the regions required for H2A.Z function are the C-terminal residues 92–102 (the αC-helix, beyond the canonical histone fold) and residues 106–119 (the distal end of the extended C-terminal tail) [14]. These regions, termed "docking regions," create a large interaction surface (2,400 Å^2^) with the (H3-H4)_2 _tetramer by guiding the interaction of the H3-αN helix with the last turn of the nucleosomal DNA, and by forming a short β-sheet with the C-terminal region of H4. From the Gaussian derived dynamics of H2A.Z (Fig. 5.I), we observed that the complete histone fold of H2A/H2A.Z is highly dynamic in the global mode (colored red) with a comparatively larger amplitude of dynamics for the αC-helix of H2A.Z, which is one of the important regions that determines the function of H2A.Z.

Whereas residues from N-terminal to the middle of α2-helix exist in a highly dynamic domain in the second slowest mode (Fig. 5.II), the rest of the histone fold expresses moderate dynamics (colored green). In comparison, the residues from N-terminal to the middle of α2-helix and the αC-helix of H2A.Z possess larger amplitude of dynamics than that of H2A (changed from orange to red color). In general, the docking region of H2A.Z (and H2A) is highly dynamic throughout the first two slow modes. Concerning the stability of the octameric histone core, it has been suggested that the L1:L1 interactions between H2A.Z monomers in the nucleosome may be responsible for the greater stability of H2A.Z-containing nucleosomes [14,39]. From Fig. 5, it is obvious that the L1:L1 interactions between the two H2A.Z monomers would be highly fluctuating due to the participation of these loops in the global dynamics. The active participation of H2A.Z in the global dynamics is assumed to play a role in the transcription activation within chromatin and also reinforces the fact that H2A.Z is preferentially associated with actively transcribed chromatin [14].

**Figure 6:**
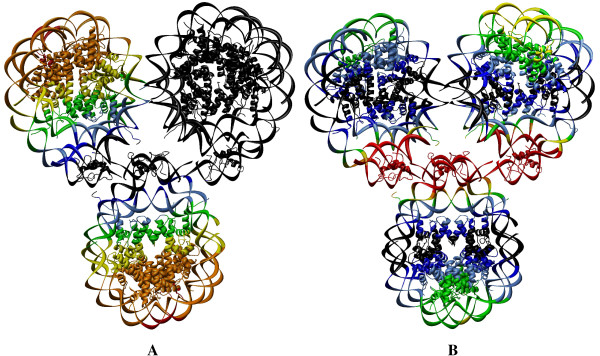
**Color-coded representation of the dynamics of the nucleosome trimer**. The color-coded diagram shows the dynamics of the 1F66 trimer in the first **(A) **and second **(B) **modes. The amplitude of fluctuation is graded the same as Figure 4.

Fig. 6 depicts the global dynamics of nucleosome trimer. The two global modes of the trimer are distinct. In the global mode (Fig. 6A), the first two nucleosomes of the complex are completely dynamic, thus similar to the dynamics of the nucleosome dimer. The third nucleosome in the first mode is highly conserved and acts as the hinge nucleosome for the free motion of the other two. The two nucleosomes express a pattern of dynamics comparable to those observed in the global mode of the dimer. In the second slowest mode, all three nucleosomes are involved in conjugate dynamics, with respect to the dyad axis, and are similar to the second slowest mode observed for the dimer. It is interesting to mention, that the trimer follows the pattern of dynamics similar to the nucleosome dimer within its global mode.

**Figure 7:**
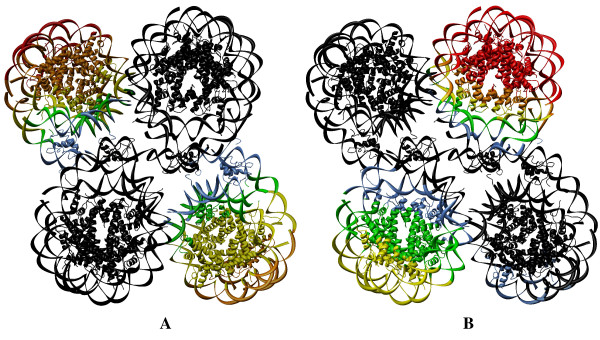
**Color-coded representation of the dynamics of the nucleosome tetramer**. The color-coded ribbon diagram shows the dynamics of the 1F66 tetramer in the first **(A) **and second **(B) **global modes. The amplitude of fluctuation is graded the same as Figure 4.

Fig. 7 depicts the color-coded diagram of the dynamics of 1F66 tetramer in the first and second slowest modes. In the first slowest mode, the two edge nucleosomes follow the first slowest global mode of the dimer whilst keeping the middle two nucleosomes rigid. In the second slowest mode, the two central nucleosomes follow the first slowest global mode of the dimer whilst keeping the two egde nucleosomes rigid. These observations reveal the role of symmetry during the global dynamics of the nucleosome tetramer in its first two slow modes. The dynamic, as well as the rigid domains, are similar to the dimer, but we have yet to explain the dynamic/rigid domains in detail for the trimer and tetramer.

The scale of our analysis is limited to only the small nucleosome complexes (up to tetramers) and is too short to explain whether H2A.Z facilitates intra nucleosome-nucleosome interactions to promote the formation of the 30nm fiber. From all the present observations, it can be stated that the incorporation of H2A.Z histone releases inter-residual interactions and thereby enhances the amplitude of dynamics in the global mode. We also reported the enhancement of histone-DNA interactions due to the incorporation of H2A.Z in our previous study. Such pronounced dynamics might be needed for nucleosome positioning [38] required to establish a specific active chromatin architecture at the promoter regions of certain genes [31]. It doesn't answer the question whether the H2A.Z generates a completely dynamic octameric core (leading to the local destabilization [39]) or forces the nucleosome into the equilibrium position (leading to the global stabilization [37]) via the incorporation of H2A.Z.

### Directionality in the global dynamics of nucleosome complexes

**Figure 8:**
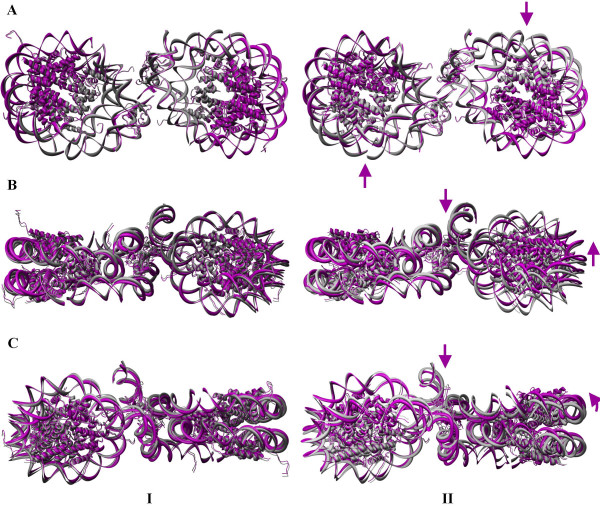
**Conformational changes during the global dynamics of the nucleosome dimer**. Superposition of the crystal structure with one conformation was generated by adding the fluctuation vector from the anisotropic fluctuation to the crystal coordinates for the dimers **(I) **1EQZ and **(II) **1F66 as shown in the front **(A) **and side views with respect to the dyad of the left nucleosome **(B) **and the right nucleosome **(C) **in the first slowest mode.

The anisotropic analysis reveals the direction of the nucleosome dynamics in the complexes. The conformational changes observed for the global dynamics of the nucleosome dimer are shown in Fig. 8. To understand the conformational fluctuation of the dimer, the figure depicts the front view (A). The views perpendicular to the dyad of each nucleosome (B for the first nucleosome and C for the second nucleosome) are also shown. The straight arrows indicate planar motions of residues, whereas the curved arrows indicate the dynamics that are perpendicular to the dyadic plane of each nucleosome. One conformation was generated by adding the ANM-derived residue fluctuations (hereafter, we will call this an ANM conformation for all complexes) and was superimposed with the crystal structure of the dimer.

Figure 8IA shows the conformational deviations observed during the global mode of the conventional nucleosome dimer. The r.m.s. value between the crystal structure and new ANM conformation were calculated by using the standard command in Chimera by fitting all atoms. The calculated r.m.s. value is 1.738 Å, meaning that both conformations are nearly identical. Superimposition of the crystal and ANM conformations showed that the nucleosome dimer expresses a conformational fluctuation perpendicular to the dyadic plane. Almost half of the nucleosomes encompassing the regions such as H3-H4 dimer and the linker DNA/histone is pushed below the crystal conformation (and hence we could see the crystal conformation colored as grey) and the other half of the nucleosome is pushed above the crystal conformation (and hence we could see the magenta ANM conformation). This leads to the bending of the nucleosome dimer perpendicular to the dyad, which is the binding site for the transcription factors [66]. Even though the GNM derived conformations revealed a highly dynamic conformation in the global mode, the amplitude of fluctuation is considerably lower for the conventional nucleosome and for this reason, no indications are given for the residual fluctuations.

Figure 8IB shows the conformational deviations observed in the global mode of variant nucleosome dimer. The H-bonding network in the dynamics of H2A.Z has been compared to the crystal structure and the ANM conformations. During the dynamics between the two ANM conformations, the two hydrogen bonds; (i) Arg 31--- G267 and (ii) Lys79 --- C278, that exist in the crystal strutcure of H2A.Z, disappear and additional H-bonds are formed. The r.m.s value between the crystal structure and new ANM conformation is 3.285 Å indicating larger deviations in the variant nucleosome and, hence, is more dynamic than the conventional one. The variant nucleosome dimer also reveals out-of-plane dynamics in the dyadic plane, yet the axis of bending is not exactly perpendicular to the dyadic axis, but is inclined ~45° to the dyad. In addition to the bending, the variant dimer also expresses notable in-plane distortions as indicated by the arrows. Such in-plane and out-of-plane motions might generate distortions in histone-DNA interactions which would weaken the stability of the nucleosome and result in nucleosome dissociation [67]. Altogether, the in-plane as well as out-of-plane motion of the conventional/variant nucleosome reveals vibrant dynamics for the variant nucleosome and is shown with linear and curved arrows. Further analysis on nucleosome complexes such as trimer and tetramer has been focused only on the variant nucleosome since the dimer has already proven the effective dynamics/influence of variant histone H2A.Z like for the variant monomeric nucleosome.

**Figure 9:**
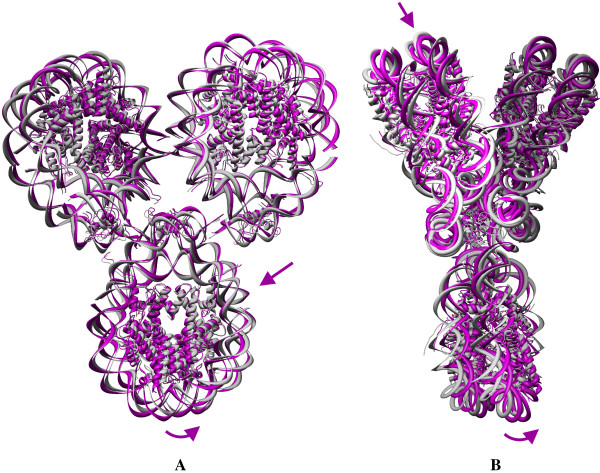
**Conformational changes during the global dynamics of the nucleosome trimer**. Superposition of crystal structure with one conformation was generated by adding the fluctuation vector from the anisotropic fluctuation to the crystal coordinates for the trimer 1F66 as shown in the front **(A) **and top **(B) **view in the first slowest mode.

**Figure 10:**
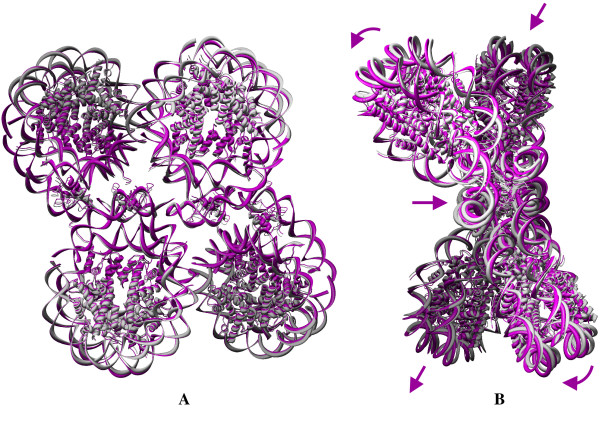
**Conformational changes during the global dynamics of the nucleosome tetramer**. Superposition of crystal structure with one conformation was generated by adding the fluctuation vector from the anisotropic fluctuation to the crystal coordinates for the tetramer 1F66 as shown in the front **(A) **and right **(B) **side view in the first slowest mode.

Fig. 9 shows the superimposition of the ANM and crystal conformation of the 1F66 trimer. Panel A shows the front view and B shows the side view in the first slowest mode. ANM reveals the dynamics of the trimer as a combination of the first two slow modes as observed from the GNM results. The global mode of the variant nucleosome trimer is similar to the dimer, as each nucleosome expresses in-plane (shown by arrows) as well as out-of-plane motions (shown by curved arrows) as observed in the dimer with the r.m.s deviation of 3.592 Å between the crystal structure and new ANM conformation. The loci of the axis separating the in-plane as well as out-of-plane lies ~45° to the dyad axis. The superimposition of new ANM conformation and the crystal coordinates for the tetramer 1F66 is shown in Fig. 10 with two different views: front (panel A) and side view (panel B). The r.m.s value between the crystal structure and new ANM conformation is 2.128 Å. In the tetramer, two nucleosomes are involved in the stretching of the nucleosome complex, and the other two nucleosomes exhibit non-planar global motions. This is similar to the conformational fluctuations derived from the dimer. Quite interestingly, the symmetry plays a role in maintaining the similar dynamics of the dimer in tetramer. Over all, our observations reveal the nucleosome dynamics through the in-planar stretching as well as out-of-planar bending motions. Recently, Roccatanao et al. studied the structural flexibility of the nucleosome core particle using molecular dynamics simulations and reported only the out-of-planar bending motion of nucleosome as one of the softest modes. However, the in-planar stretching and compression dynamics of nucleosome we observed as one of the global relaxation dynamics has not been explained by any other groups [68]. Another rather new observation contained in this work is the combination of the relaxation mechanisms adopted by the individual nucleosomes in the complex; furthermore, this may lead to further insights about the mode of relaxation of nucleosome complexes.

During the dynamics of all these complexes, the interaction between the histone octamer and duplex DNA is highly altered and is expected to accompany a change in the position of the octameric histone, with respect to the DNA, playing a main role in nucleosome sliding [21,69,70]. Through the dynamics, the region encompassed by the linker histone and DNA are not very flexible – possibly due to the binding of the linker histone and linker DNA region. Incorporation of histone H1 into nucleosomal complexes results in essentially inactive chromatin templates with respect to both transcription elongation and initiation [71].

## Conclusion

The normal mode analysis of the global dynamics of nucleosomal complexes such as dimer, trimer and tetramer with conventional as well as variant H2A.Z histones has been studied using normal mode analysis. Dissection of the dynamics of individual nucleosomes in the complex discloses in-planar and out-of-planar fluctuations as the common mode of relaxation throughout the global motions, but the loci of the axis characterizing out-of-plane motions deviate irrespective of the number of nucleosomes present in the complex. Distortions generated by the non-planar dynamics also influence the DNA sequences and hence the histone-DNA interactions significantly alter the dynamics of the DNA, conversely influencing the transcriptional activity of the nucleosome as well as chromatin [67].

The presence of variant histones highly influences the dynamics of the nucleosome complexes. Comparison of the dynamics of variant H2A.Z with conventional H2A histone reveals that the complete histone fold of H2A and H2A.Z is dynamic in the global mode with enhanced dynamics at (i) the αC-helix of H2A.Z, and (ii) the residues encompassing the L1:L1 interaction regions between H2A.Z monomers. However, only half of the histone fold (the residues from N-terminal to the middle of α2-helix) exists as the highly dynamic domain in the second slowest mode. The highly dynamic/active variant H2A.Z is assumed to play a part in the transcription activation within chromatin. Hence, it can be concluded that the highly dynamic H2A.Z histone is a major source of weaker intramolecular and intermolecular couplings/correlations [1,36].

The pronounced fluctuation of H2A.Z that leads to the enhanced dynamics of the nucleosome complexes is also expected to influence the folding of H2A.Z reconstituted chromatin unlike that of the H2A chromatin [44]. Experimentally, the replacement of H2A.Z for H2A histone in cells induces a shift in the positioning of a nucleosome located over the transcription start site [39]. However, it is essential to determine whether the simple incorporation of H2A.Z into the nucleosome is sufficient to facilitate gene activity. The higher dynamics of the nucleosome complex is assumed to participate in the disruption of the nucleosome due to the higher mobility and the disorder of the variants. Hence, *in vitro*-assembled chromatin complexes containing H2A.Z are more resistant to condensation when compared to H2A-containing nucleosome complexes [43,44]. The enhanced dynamics of H2A.Z single and oligo-nucleosomes is in agreement with their positioning in the gene promoter area and their related role in gene regulation [44,72]. Nucleosome positioning correlates with the positioning of other promoter elements (core promoter elements and binding sites of specific transcription factors) [73-76]. Promoter chromatin structure should be remodeled to facilitate the transcription. The dynamic nature of the H2A.Z nucleosomes makes them more susceptible to this remodeling. The consistency in the dynamics pattern of the nucleosome complexes observed in the present study highlights the existence of nucleosome in various relaxation modes and the role of variant histones over the global dynamics.

Even though the nucleosome expresses the two major relaxation mechanisms [(i) in-planar motions and (ii) out-of-planar bending motions], it is very interesting to show how the individual nucleosome selects/adopts the relaxation mechanism when the dynamics of the whole nucleosome complex is considered. The choices/combination of the relaxation mechanisms adopted by the individual nucleosomes in the complex are also rather new and these observations could enrich the knowledge of the relaxation modes of nucleosome arrays (nucleosome complexes having more than six nucleosomes) when the analysis is extended beyond the order of the nucleosome complexes.

## Methods

Coordinates of the linker histone H1 were taken from the monomer structure of the PDB: 1GHC[77]. A linear B-DNA fragment of length 19bp was constructed using the Biopolymer module of the Insight II package. A complete nucleosome monomer with which the nucleosome complex was modeled was built using a nucleosome of the PDB database: 1F66 for variant nucleosome complex and 1EQZ for normal nucleosome complex. At first, two B-DNA fragments of 10 bp (base pairs) with the same sequence on both terminus of the nucleosome were modeled using the biopolymer module of Insight II and superimposed on each DNA terminus of the nucleosome. After superimposing, the newly built 10 bp were connected at the arms of the nucleosome crystal structures. The existing 10mers were removed, as these sequences possess deformed base stacking and may not extend without steric interaction for any order of nucleosome polymerization. Secondly, a B-DNA of sequence 5'-CTGCAGATTCTACCAAAAG-3' was modeled and extended at both ends to form the linker DNA region. At this stage, the nucleosome can interact with the linker histone H1 with its helix bundle. The linker histone was then located, so it would make the hydrogen bonds with the duplex of the DNA [16,17]. After constructing the monomer, the latter was allowed for structural minimization by using the steepest descent method to attain the globally stable conformation. After optimization, the monomer was used to model the nucleosomal complexes as the "beads on a string" model. The configuration of nucleosome complexes of regular H2A histone and the variant H2A.Z histone are modeled using the crystal structures 1EQZ[78] and 1F66[14] respectively. The nucleosome 1F66 corresponds to the recombinant mouse H2A.Z and recombinant *Xenopus leavis *H2B, H3 and H4, whereas 1EQZ refers to the chicken (*Gallus gallus*) histone octamer. Despite the differences in the originating organisms, the nucleosomes possess high (>95%) sequence identity with the exception of the variant histone H2A.Z with the r.m.s. deviations between α-carbon coordinates of ~0.50 Å (see [1] for more details). All the nucleosome complexes were modeled using the molecular modeling package Insight II. Recently, Schalch *et al*. [63] reported the crystal structure of a tetranucleosome of *Xenopus leavis *origin at 9 Å resolution as solved by molecular replacement using the nucleosome core structure (PDB id: 1ZBB). We used this conformation to validate the modeled complexes and to exhibit a comparison between inter- as well as intra- domain motions of the model dimer (see Results and Discussion for examples).

In the Gaussian network model (GNM), the elastic network is constructed by considering the α-Carbon atoms of each residue as nodes connected by the elastic spring constant γ. The interaction sites within a spherical shell of *r*_*c *_are considered coupled harmonic oscillators subjected to Gaussian fluctuations around their mean position. The Hamiltonian of the conformation is derived from the Kirchhoff matrix of contacts (Γ) by considering local packing density (pairs of amino acid or nucleotide residues within the cutoff distance, *r*_*c*_) and coordination order (the separation of the contacting residues along the backbone). In proteins, the C_α _network is considered with *r*_*c *_≤ 10 Å. DNA, as well as RNA, has a slightly increased cutoff distance ~14 ± 2 Å to incorporate inter-strand interactions [79-81]. The Kirchhoff matrix, Γ, is an *N *× *N *symmetric matrix for a sequence of *N *residues with values of -1 for contacting i and j residues, and 0 for non-contacting residues. The diagonal elements are obtained using the expression Γii=−∑jΓij
 MathType@MTEF@5@5@+=feaafiart1ev1aaatCvAUfKttLearuWrP9MDH5MBPbIqV92AaeXatLxBI9gBaebbnrfifHhDYfgasaacPC6xNi=xH8viVGI8Gi=hEeeu0xXdbba9frFj0xb9qqpG0dXdb9aspeI8k8fiI+fsY=rqGqVepae9pg0db9vqaiVgFr0xfr=xfr=xc9adbaqaaeGacaGaaiaabeqaaeqabiWaaaGcbaGaeu4KdC0aaSbaaSqaaiabdMgaPjabdMgaPbqabaGccqGH9aqpcqGHsisldaaeqbqaaiabfo5ahnaaBaaaleaacqWGPbqAcqWGQbGAaeqaaaqaaiabdQgaQbqab0GaeyyeIuoaaaa@39DB@ where j ≠ i. In GNM, motions are expressed as a superposition of modes of different frequencies. The coarse grained dynamics of the nucleosome complex is decomposed into a collection of *N*-1 internal modes with frequencies λ_k_, 2 ≤ k ≤ *N*.

An effective elastic network for the nucleosome dimer is generated using a cutoff distance *r*_*c *_≤ 10 Å for protein, 16 Å for the DNA and the interaction between DNA and protein. In the case of the trimer, the interaction between each pair of protein residues uses the distance *r*_*c *_≤ 14 Å and 20 Å for the DNA and protein-DNA interactions. For the tetramers, the cutoff distances 40 Å and 60 Å are used for proteins, DNA, and protein-DNA interactions.

ANM is an extension of GNM, where the anisotropic fluctuations from the second derivative of harmonic potential of residues are considered. Fluctuation vectors (Δ**R**_i_) in the Cartesian coordinate frame [56] are generated. The three components of the fluctuation vector help to find the action of individual modes in alternative conformations of the biomolecular complex. This is done by incorporating the vector ± Δ**R**_i _with the equilibrium vectors. For this analysis, the networks for 1F66 dimer and trimer were constructed using the cutoff distances 30 Å, 35 Å and 40 Å for the protein, DNA, and protein-DNA respectively. For the 1EQZ dimer, the cutoff distance is 35 Å for protein, 40 Å for DNA, and 45 for DNA-protein interaction. The network for 1F66 tetramer was built using a cutoff distance of 40 Å for protein, 45 Å for DNA, and 50 Å to include the DNA-protein interaction. All the resulting matrices (Hessian matrix) of size 3*N *× 3*N *were solved for the eigenvalue and eigenvectors for analysis. Given that the slowest modes refer to the most cooperative motions between entire subunits or subdomains [82-84], focus is only paid to the slowest modes for both GNM and ANM analysis.

The calculations were performed using a desktop PC for ANM/GNM modeling of single nucleosomes and dinucleosomes, and a Cray X1 of the Ohio Supercomputer Center for tri- and tetranucleosomes. Supplemental modeling performed using SGI Octane 2 workstation. From computational time point of view, the eigenvalue decomposition of the connectivity matrix **Γ **is the most expensive task in GNM calculations. The singular value decomposition (SVD) subroutine [85] used in GNM scales up the computing time with *N*^3 ^for a network of *N *residues. For *N *< 2000, the computations are performed within minutes.

## Authors' contributions

AR performed major calculations and participated in drafting the manuscript. II conceived of the study and directed the work, including its design and coordination, drafting the manuscript and editing it. All authors read and approved the final manuscript.
